# The University of Maryland

**Published:** 1883-09

**Authors:** 


THE UNIVERSITY OF MARYLAND.—The Second Annual Session of the Dental Department of this University will begin under the most favorable auspices on the first day of October next. Already, although almost a month is yet to elapse before the regular or winter session begins, the number of matriculates is quite large, and there is every indication that the class for the coming session will be an unusual one for size. The encouragement from the honored and respected members of the dental profession throughout the country which this Institution is receiving, is very gratifying, and its success is well assured. The unprecedented class of last session was an evidence of the appreciation of the many advantages which such a Dental Department offers over separate schools. The Infirmary and Laboratory practice has attained such a size that abundance of materials for the practical instruction of dental students is assured for the future beyond doubt.

The name of Dr. G. F. S. Wright, of Columbia, South Carolina, was inadvertently omitted from the list of Clinical Instructors as published in the last Catalogue, and as Dr. Wright is a member of this Corps of the University of Maryland, we deem it due to him to thus announce the fact in this JOURNAL.

The Law School of the University is now erecting on the University grounds, a fine and commodious building for their department, and with its completion the University will have all the buildings of its different departments in one locality, and on its own land. The University grounds around the different structures are to be improved and beautified in such a manner as will render them very attractive, and present a pleasant view to the numerous patients of the extensive University Hospital located on the opposite square.

